# A Theoretically Informed Process Evaluation in Parallel to a Feasibility Study of a Complex Oral Health Intervention Using NICE Guidelines in a Care Home Setting

**DOI:** 10.1111/cdoe.13016

**Published:** 2025-01-15

**Authors:** Paul Brocklehurst, Joe Langley, Rebecca Wassall, Sana Daniyal, Saif Sayeed Syed, Michelle Harvey, Nia Goulden, Andrea Sherriff, Anja Heilmann, Zoe Hoare, Craig Smith, Richard Watt, Ciaran O'Neill, Frank Kee, Peter Cairns, Nat Lievesley, Gerald McKenna, Georgios Tsakos

**Affiliations:** ^1^ Public Health Wales Cardiff UK; ^2^ Lab4Living, Sheffield Hallam University Sheffield UK; ^3^ School of Dental Sciences Newcastle University Newcastle UK; ^4^ Institute of Epidemiology & Health Care University College London London UK; ^5^ Centre for Public Health Queens University Belfast Belfast UK; ^6^ North Wales Medical School Bangor University Bangor UK; ^7^ School of Medicine University of Glasgow Glasgow UK; ^8^ Division of Cardiovascular Sciences University of Manchester UK; ^9^ PPI Contributor UK

**Keywords:** care home, oral health, process evaluation, reflexive workshop, systems lens

## Abstract

**Background:**

A theoretically informed process evaluation was undertaken in parallel to a study examining the feasibility of an oral health intervention based on an existing guideline for care homes. The objectives were to explore the factors that influenced the implementation of the intervention in order to understand the potential pathway to impact. The research team initially utilised Pfadenhauer et al.'s framework, which focuses on a number of different implementation factors: intervention characteristics, context, theory, process, strategy, agents, outcomes and setting.

**Methods:**

Nine semi‐structured interviews were undertaken with care home managers and staff, predominantly within the intervention arm of the study. Interview schedules were originally based on Pfadenhauer et al.'s framework. These were coded and analysed using thematic analysis. Given the range of themes that emerged, the research team ran a reflexive workshop to determine whether Pfadenhauer et al.'s framework was able to capture and frame the authentic voice of those interviewed.

**Results:**

The research team found that a systems lens approach better fitted the data from the interviews, capturing the idiosyncrasy of the different settings and the importance of values and beliefs of the key stakeholders. It was clear that unlike the structure proposed by Pfaednhauer et al., many of the factors were interdependent and hierarchical in nature, that is, paradigm and goals within the care home had a direct impact on the system structure, which fed into how the care home was maintained, which led onto how the different actors (care home managers and staff) behaved. The process also highlighted key factors for intervention delivery: time poverty, competing needs, staff turnover, differences between shift patterns and between permanent and agency staff. Cognitive capacity of the residents and staff attitudes were also key.

**Conclusions:**

Adding a reflexive workshop enabled the research to critically review the Pfadenhauer et al.'s framework and change to a systems lens approach, which better explained the interdependent and hierarchical nature of the findings. It also highlighted a number of key factors that could influence the pathway to impact for the intervention.

**Trial Registration:**

ISRCTN10276613

## 1 Background

Oral health in care homes is poor, with residents experiencing two to three times the level of dental disease when compared to their community‐dwelling peers ([1]). The National Institute for Health and Care Excellence (NICE) guidelines ‘Oral health for adults in care homes (NG48)’ were issued in 2016 with the aim to maintain and improve the oral health of care home residents [2]. However, the available literature to inform the guidelines was relatively sparse, with few empirical studies [2]. Equally, the NG48 recommendations only had a limited number of concrete actions that could be utilised by care home staff. As a result, a feasibility study entitled ‘Improving the Oral HealTh of Older People In Care Homes (TOPIC)’ was undertaken to determine whether a definitive randomised controlled trial was potentially possible [1]. TOPIC was a pragmatic cluster randomised feasibility study with a 12‐month follow‐up that was undertaken in 22 care homes across two sites (11 in London, 11 in Northern Ireland). Care homes randomised to the intervention arm received a complex intervention based on the NG48 guidelines, which had been subsequently refined using a ‘codesign’ method with care home managers and staff [3]. The intervention was composed of a training package for care home staff to promote knowledge and skills in oral health promotion, the use of an adapted Oral Health Assessment Tool to assess oral health needs of the residents, a Personal Oral Care Plan, a weekly Oral Hygiene Record and a ‘support worker assisted’ daily toothbrushing regime with toothpaste containing 1500 ppm fluoride. Care homes in the control arm were to continue according to their usual routine, so did not receive any of the intervention materials.

In parallel to the feasibility study, a theoretically informed process evaluation was undertaken to explore the context and the types of factors that could impact implementation. The importance of accounting for the setting has recently been advocated by the Medical Research Council, who argue that context and practice should be considered equally important components in the implementation of evidence‐based interventions [4]. In this sense, organisation features, resources, the nature and quality of leadership, the prevailing culture and the communication systems within a given setting are all key [5, 6].

This paper describes an approach that started with the use of the Pfadenhauer et al. [6] framework to address the objective of the study to ‘undertake a parallel process evaluation to explore how the intervention could be embedded in standard practice’ [1]. However, after reviewing the data and running a reflexive workshop, our approach was adapted in light of the emerging themes to adopt a systems lens approach. Pfadenhauer et al. [6] is a framework that focuses attention on the following aspects of implementation: intervention characteristics, context, implementation theory, implementation process, implementation strategy, implementation agents, implementation outcomes and setting. Systems lens approaches hold a more reflexive and pluralistic view of knowledge mobilisation, which argues for an understanding of how this knowledge moves around systems and how it is constrained or gains legitimacy via system structures, actors, resources and the dynamics therein [5]. It also places elements within a hierarchy, where the explicit or implicit beliefs that make a system work (‘paradigm’) are seen as most important and impact on the aim and purpose of the system (‘goal’). This in turn acts upon the regulation and organisation of the system (‘system structure’) to maintain the status quo (‘feedback’) and the stakeholders and physical resources that form the discrete parts of the system (‘system elements’) [5]. The aim of this paper was to present the main findings of the process evaluation and how the research team used the reflexive workshop to restructure the approach taken.

## 2 Methodology

The study received ethical approval from the London City & East Research Ethics Committee (19/LO/1107). To inform the process evaluation, the research team initially drew up a set of questions related to the key findings from the intervention refinement phase [3], which were then mapped onto the Pfadenhauer et al. [6] framework (Table 1). The process evaluation was undertaken during the active phase of the feasibility study (12 months).

**TABLE 1:** Interview questions mapped onto Pfadenhauer *et al*., [6] framework.

Domain in Pfadenhauer et al.	Detailed question
Intervention characteristics	How important is oral health in a care home setting?
	What did you think to the Oral Health Assessment Tool?
	Anything in the intervention that you would modify, add or remove?
	Any tensions between the intervention and what the residents’ personal preferences were?
	Any competing needs with other care requirements?
Context	Did the staff have the time to deliver the intervention fully?
	Did your staff feel confident in using the materials?
	What do you think of the care home staff training package? Was it appropriate? Anything that you would change or modify?
	Did you have to change the intervention in any way to accommodate the resident's changing cognitive ability/needs? Was the intervention flexible enough?
	Any refusal behaviours? How did the staff manage these?
	Any dangers to staff when implementing the intervention (e.g., as a result of refusals)?
	Any coping strategies that the staff had to use when things didn't work?
Implementation theory	We designed the intervention with care home staff, did this help? What were your thoughts about the intervention?
Implementation process	Do you think the intervention met the needs of the residents?
	Do you think you have enough resources, such as staff or time to implement and maintain this intervention? Would you like to have more support?
	Did your staff feel confident in using the materials?
	What do you think of the care home staff training package? Was it appropriate? Anything that you would change or modify?
Implementation strategy	Questioned above
Implementation agents	What did you think to the Oral Health Assessment Tool?
	Anything in the intervention that you would modify, add or remove (Personal Oral Care Plan, Weekly Oral Hygiene Record, Tips and Tricks, Oral Health Poster)?
	Any tensions between the intervention and what the residents’ personal preferences were?
	Did the staff have the time to deliver the intervention fully?
	Any competing needs with other care requirements?
	Did you have to change the intervention in any way to accommodate the resident's changing cognitive ability/needs? Was the intervention flexible enough?
Implementation outcomes	Did involvement in TOPIC cause any difficulties or problems in the general day‐to‐day running of your home?
	Thinking about your home's involvement in the TOPIC study was the experience positive? Can you give me an example?
	Anything negative?
	How did the staff cope with the form filling? Any difficulties completing the weekly checklists and OIDPs?
Setting	Questioned above

The data collection for the process evaluation started with members of the research team (SD, SSS and MH) approaching care home staff in both the intervention and control groups, ahead of undertaking the interviews. In accordance with a process evaluation aimed at understanding the factors that could influence the implementation of the intervention, all participating care homes engaging in the intervention group were approached, including care home managers and staff (although the latter were often too busy to be interviewed). Residents were also approached but most declined to participate and the one interview that proceeded highlighted how levels of cognitive impairment impacted on the understanding of the intervention. A number of care homes in the control arm were also approached for interviews to identify any further factors that may be pertinent to the context of providing care. Those potentially interested in taking part were provided with a Participant Information Sheet and were given 48 h to decide whether they wished to take part. The types of stakeholders that were interviewed for the study are provided in Table 2.

**TABLE 2:** Participants that were interviewed for the process evaluation.

#	Stakeholder role	Geographic location
1	Care home manager (intervention group)	London
2	Care home manager (intervention group)	London
3	Care home manager (intervention group)	London
4	Care home manager (control group)	London
5	Care home staff (intervention group)	London
6	Care home staff (control group)	London
7	Care home resident (control group)	London
8	Care home manager (intervention group)	Northern Ireland
9	Care home manager (intervention group)	Northern Ireland

The semi‐structured interviews lasted between 30 and 60 min and were digitally recorded. Participants were provided with the option to have the interview conducted in person at the care home, by telephone or using a digital platform. The interviews were guided by a schedule based originally on the Pfadenhauer et al. framework, focusing on the following factors: intervention characteristics, context, theory, process, strategy, agents, outcomes and setting. Given the COVID pandemic, all the care home staff requested a remotely held interview via Teams. As such, the research team utilised the transcription function of Teams to provide MS Word documents. Interviews were then analysed and mapped into the theoretically informed framework for the process evaluation using a thematic approach.

To conduct the analysis of the interviews, three members of the research team (SD, SSS and MH) immersed themselves in the data by initially reading and re‐reading the transcriptions. This process was overseen by PRB and subjectivity was reduced as far as possible through reflexivity and the use of multiple coders (SD, SSH, MH and PRB). Quotes were then compared with the theoretical framework and recorded in an Excel document, that acted as a master file. It was determined in advance that the interviews would continue until saturation had been reached [7]. This was assessed by PRB, when no new information was generated from the interviews deductively and when no further additional themes/codes were elicited inductively that were not initially within the Pfaednhauer et al. framework.

Towards the end of the process evaluation, members of the research team (GT, PRB, JL, RRW, SSS, SA and MH) undertook a reflexive workshop to review the findings and the applicability of the Pfadenhauer et al. [6] framework for a care home setting. Data were collected to understand the experience of the researchers in undertaking the interviews. At the workshop, the following factors were explored:

1. Appropriateness of the questions and whether the collected data reflected what they were sensing and experiencing from working in the care homes; and
2. Identification of any key aspects of the findings that were not easily mapped into the Pfadenhauer et al. [6] framework

The findings from the interviews and the workshop led the research team to reconsider the usefulness of the Pfadenhauer et al. [6] framework. As such, the interviews and workshop findings were combined and analysed using a hierarchical system lens approach to produce a ‘meta’ and more reflexive view of the narrative [5].

## 3 Results

Care home managers and care home staff from six different care homes in the intervention arm were interviewed, along with three of the 11 care homes that were allocated to the control arm. Time pressures during the study period (which coincided with the COVID‐19 pandemic) meant that no other care homes were able to participate in the interviews.

As highlighted above, the coding of the transcripts initially followed the framework from Pfadenhauer et al. [6], however, what became apparent when reviewing the data was that the framework was not describing the key phenomena that were emerging. Focusing on the intervention characteristics, context, implementation theories, processes, strategies, agents, outcomes and settings, did not account for how these were often interdependent in care homes and how they acted upon another in a hierarchical manner. For example, key stakeholders were able to influence the delivery of the intervention in substantive ways. In many homes, this was the care home manager, but it could also be a member of staff who had a real interest in oral health. Following the reflexive workshop, where these issues were surfaced we changed our framework to a system lens approach and recoded accordingly.

The findings are presented according to the key leverage points in the hierarchical system lens approach, which formed the new framework for the elicited codes (Table 3). Interview and workshop quotes are prefaced with ‘I' and ‘W' respectively. Further detail is available in the Supporting File.

**TABLE 3:** System lens applied to interviews and workshop data.

Leverage points	Description	Codes from interviews and workshops	Explanation of codes
Paradigm and goals	Values and explicit/implicit beliefs that makes the system work and aims to deliver to this system mindset	Desire to improve oral health and participate in an oral health intervention	Importance of oral health and oral health routine
System structure	Elements that make up the system (subsystems, actors, elements and their interconnections)	Idiosyncrasy of care homes	Care homes are highly variable (e.g., values, beliefs and structures)
		Position and personality of the person running TOPIC	Authority, personality and drive of the person responsible for delivering the intervention in the care home
		Time poverty	Lack of time of care home staff to deliver the intervention
		Competing needs	The additional burden of providing an oral health routine in a busy care home
		Different shift patterns	Influence of different shift patterns on the culture and preparedness to provide an oral health routine
		Staff turnover	The impact of regular staff turnover on maintaining continuity of an oral health routine
		Permanent and agency staff	The impact of using agency staff and the loss of knowledge from the system
Feedback	System regulation and organisation to maintain stability and status quo (e.g., through power and roles)	Excessive paperwork	Documenting activity and the additional burden of recording the details of an oral health routine
		Rhythm and processes	Importance of aligning any new oral health regime with the existing rhythm and processes of the care home
		Challenge of training	The importance of training in establishing a minimum level of knowledge to support an oral health routine
Structural elements	Stakeholders and physical resources that form the discrete parts of the system	Intervention acceptability	How well the intervention was received by the resident
		Cognitive capacity of the resident	The influence of cognitive capacity on the oral health routine
		Staff attitudes and confidence	Attitudes towards the oral health routine and confidence in determining the difference between oral health and disease
		Intervention components	Details of the different components of the oral health routine

### 3.1 Paradigm and Goals

All the care home managers interviewed expressed a strong desire to participate in the study, particularly as access to local dental services was difficult.

There's more coming in with their own teeth [AND] their own teeth are in such a state. Sometimes it's only plaque that's holding the teeth together I:8.14

Equally, participation in the study appeared to have a marked impact on the reported daily routines for care home staff.

We knew that they weren't really brushing their teeth as often as they said. So now they're really on board and they point out to you after breakfast, I'm away to brush my teeth now I:8.40 (care home manager)

### 3.2 System Structure

The manner of adoption of the study appeared to be relatively idiosyncratic and the organisation and the efficiency of processes within each home were very different.

They're so different in their structure. The context impacts whether the intervention works and how they respond to it W:2,887 (researcher 3)

Equally, certain features of the care home were seen to be more important than others. One key element here was the position and personality of the person in charge of dealing with the TOPIC study at the home.

It's just really the personality of the manager. If you have a really good, enthusiastic manager, that's it W:720 (researcher 3)

Junior care home staff would struggle to make it happen because they're not high enough in the hierarchy in that particular care home…. W:2,936 (researcher 1)

Time poverty was raised as a significant issue in the workshop and in the interviews.

These people are so over worked. They are very well motivated. They want to do the study. It's just they don't have the time to do that W:320 (researcher 1)

Competing needs was another factor raised in the interviews, with care home staff prioritising one task over another due to time poverty and the fluctuating care needs of the residents. The new oral health routine was an additional task in an already busy schedule, and this was exacerbated further in homes with a higher proportion of residents requiring nursing care.

So, they are more happy to give someone a shower and give them breakfast and forget about those other bits, yeah I:1.862 (care home manager)

Differences between shift patterns, shaped by the routines within the care home was also raised in the interviews and the workshop.

It's done in the morning. They wake them up, take them to the bathroom, change them, wash them…. ….at night it's not the same W:1,860 (researcher 1)

Staff turnover was an important factor raised in both the interviews and the workshop.

Every week we have new team members I:5.292 (care home manager)

Equally, there appeared to be a difference in how permanent and agency staff responded to the study in a number of homes.

Staff wise…. ….it really affected us greatly because when we started most of the team members that we have were agency I:5.292 (care home manager)

### 3.3 Feedback

One key element that arose at this level was the issue of excessive paperwork and some of those interviewed felt that this should have been integrated more with the residents' Care Plans.

It's just the paperwork. Because in their mind the last thing they're thinking of is to fill up to do paperwork I:6.651 (care home staff)

Equally, many of those interviewed expressed the importance of integrating anything new, like an oral health routine, into the existing rhythm and processes of the care home.

But if I was doing it today, I would actually make sure the forms were in their bedroom, because that's where everything takes [place] I:9.176 (care home manager)

Care home managers often cited the challenge of training in a care home environment, which was exacerbated by the level of staff turnover.

I've managed services before. It is quite difficult. Because you've got to have the knowledge yourself to share that knowledge I:1.106 (care home manager)

### 3.4 Structural Elements

Most residents appeared to receive the intervention well, but the relationship with the care staff appeared key.

It's about getting to know your resident, what works, what doesn't work I:1.1,512 (care home manager)

The cognitive capacity of the resident was also viewed as important.

And as much as I want to be able to communicate…. ….every senior is challenging I:5.470 (care home manager)

In the reflexive workshop, cognitive capacity was also reported to fluctuate over the course of the study, which could impact on communication and make it very difficult at times to collect the research data.

The cognitive impairment had got worse. And then we're asking questions. It's so difficult for them to understand and answer W:1,972 (researcher 1)

Staff attitudes also varied and not all wanted to participate in an intervention to improve oral health.

There are quite a few [staff] that don't wanna take part. I'll be honest with you. I'm the only one I:2.1162 (care home staff)

Staff felt that the Oral Health Assessment Tool was helpful as it raised the awareness of oral health within the care home.

I'm getting to understand who actually brushes their own teeth. Who's having a challenge with it? 1.138 (care home manager)

## 4 Discussion

The findings of the process evaluation highlighted the complexity of introducing an oral health intervention in a care home setting. The organisation and the efficiency of processes within each home were very different. Time poverty was a significant issue and competing needs were another factor that was seen as important. Staff turnover and the differences between different shift patterns were seen as influential, as was the difference in how permanent and agency staff responded to the intervention. Finally, the cognitive capacity of the resident and staff attitudes towards the use of an oral health intervention varied considerably.

There have been recent calls to use a complex systems perspective to public health interventions, given that many studies ‘describe static systems at a single time point’ [8]. Our findings concur with this call. Changing the underlying theoretical framework from Pfadenhauer et al. [6] to a system lens approach highlighted the hierarchical structure of the different ‘systems’ within the setting. Unlike Pfadenhauer et al. [6], where the different components of implementation are given equal weighting, the beliefs and values of those who owned or ran the care home were seen as key in making the day‐to‐day processes in the home ‘work’. The ‘paradigm’ that this produced was then critical to how the goal, structure and feedback mechanisms were organised and how the discrete elements of the system were orchestrated, including the implementation of the oral health intervention. Equally, the beliefs and values of those that were delivering the intervention ‘on the ground’ also appeared critical to the delivery of the intervention. In other words, beliefs and values appeared important in how the TOPIC materials were applied in practice. These findings would suggest that at both the level of setting the ‘paradigm’ for the care home and in the delivery of the intervention in context and in practice (‘on the ground’), there was a degree of emotional and subjective involvement in judgements and decisions that led to differences across the care homes.

These findings are consistent with recent studies [3, 9, 10, 11]. Patel et al. [10, 11] reported that the provision of oral care was a challenging task for many staff and Johnson et al. [9] argue that oral health routines are highly personal. This is important given that many residents describe a loss of identity as they lose independence [12]. Preserving residents' sense of dignity is key and oral health plays a key role in this [9, 13]. As cognitive function becomes impaired, this only adds to the challenge and can lead at times to even aggressive behaviour on behalf of the residents [3]. Given that many residents are entering care homes with increasing care needs, this presents a particular challenge for future oral care and for the implementation of ‘rigid’ guidelines to be adopted by staff [11]. The level of training needed for staff and the opportunity cost of undertaking an oral health intervention, as opposed to another care routine, have also been highlighted [10]. The constant flux between agency and permanent staff only adds to this challenge [14].

The use of a workshop added value to the process evaluation and enabled the research team to reflect on the main issues identified and determine the suitability of the analytical framework. The change to a systems lens approach made a marked difference to the coherence of the narrative. The appreciation of a hierarchical structure of influence and the importance given to the values and beliefs of both those who set the paradigm within the home and those that undertake the work ‘on the ground’ was pivotal to gaining a better understanding of how the TOPIC materials were being implemented. The findings would appear to show that an intervention must fit with the values and beliefs of those in the system, align cognitively with their ‘technical’ knowledge and be practically embedded within a given context [15]. While there are examples of using a system lens in the literature, the importance of values and beliefs is largely ignored by many implementation frameworks. In this setting, this appears to be key.

In summary, this study showed the granularity and range of factors that can influence the implementation of an oral health intervention in a care home environment. Even though a codesign approach had been taken to the development of the TOPIC materials [3], its implementation was idiosyncratic to each individual care home. The findings from this study highlight the need to consider adaptability at an organisational level, that is, that core and peripheral dimensions of interventions are in turn, influenced by the different levels within the care home ‘system’. In many senses, this is not dissimilar to the idea of ‘mindlines’, where interventions work best if they ‘fit’ the embodied forms of knowing within dynamic ‘practices‐in‐context’ [16].

## 5 Conclusion

This process evaluation found a range of factors that influenced the implementation of an oral health intervention in a care home environment. The use of a systems lens enabled the research team to account for the hierarchical structure of influence in these settings. Equally, the importance of values and beliefs were seen as key.

## Author Contributions

G.T., G.M.K. and P.R.B. conceived the study and together with Z.H., R.G.W., R.R.W., A.S., C.J.S., F.K., C.O.N., A.H., P.C. and N.L. were responsible for the study design. P.R.B. and G.T. oversaw the process evaluation and the reflexive workshop with J.L. and R.R.W. P.R.B., G.T., J.L. and R.R.W. prepared the first draft of the manuscript and revised it following input from the whole team.

## Conflicts of Interest

The authors declare no conflicts of interest.

## Supporting information


Supporting File S1.

## Data Availability

The data that support the findings of this study are available on request from the corresponding author. The data are not publicly available due to privacy or ethical restrictions.

## References

[1] G. Tsakos , P. R. Brocklehurst , S. Watson , et al., “Improving the Oral Health of Older People in Care Homes (TOPIC): A Protocol for a Feasibility Study,” Pilot and Feasibility Studies 7, no. 1 (2021): 138. 34215322 10.1186/s40814-021-00872-6 PMC8249429

[2] National Institute for Health and Care Excellence , Oral Health for Adults in Care Homes (NG48) (Manchester: National Institute for Health and Care Excellence, 2016).

[3] J. Langley , R. Wassall , A. Geddis‐Regan , et al., “Putting Guidelines Into Practice: Using Co‐Design to Develop a Complex Intervention Based on NG48 to Enable Care Staff to Provide Daily Oral Care to Older People Living in Care Homes,” Gerodontology 40, no. 1 (2023): 112–126. 35288971 10.1111/ger.12629

[4] K. Skivington , L. Matthews , S. A. Simpson , et al., “Framework for the Development and Evaluation of Complex Interventions: Gap Analysis, Workshop and Consultation‐Informed Update,” Health Technology Assessment 25, no. 57 (2021): 1–132, 10.3310/hta25570. PMC7614019 34590577

[5] A. Haynes , L. Rychetnik , D. Finegood , M. Irving , L. Freebairn , and P. Hawe , “Applying Systems Thinking to Knowledge Mobilisation in Public Health,” Health Research Policy and Systems 18, no. 1 (2020): 1–9. 33203438 10.1186/s12961-020-00600-1 PMC7670767

[6] L. Pfadenhauer , A. Gerhardus , K. Mozygemba , et al., “Making Sense of Complexity in Context and Implementation: The Context and Implementation of Complex Interventions (CICI) Framework,” Implementation Science 12 (2017): 21. 28202031 10.1186/s13012-017-0552-5 PMC5312531

[7] V. Braun and V. Clarke , “Using Thematic Analysis in Psychology,” Qualitative Research in Psychology 3 (2006): 77–101.

[8] E. McGill , D. Marks , V. Er , T. Penney , M. Petticrew , and M. Egan , “Qualitative Process Evaluation From a Complex Systems Perspective: A Systematic Review and Framework for Public Health Evaluators,” PLoS Medicine 17, no. 11 (2020): e1003368, 10.1371/journal.pmed.1003368. 33137099 PMC7605618

[9] I. G. Johnson , M. Z. Morgan , and R. J. Jones , “Oral Care, Loss of Personal Identity and Dignity in Residential Care Homes,” Gerodontology 40 (2022): 200–206. 35445763 10.1111/ger.12633

[10] R. Patel , C. Robertson , and J. E. Gallagher , “Collaborating for Oral Health in Support of Vulnerable Older People: Co‐Production of Oral Health Training in Care Homes,” Journal of Public Health 41, no. 1 (2019): 164–169. 29186499 10.1093/pubmed/fdx162

[11] R. Patel , R. Fitzgerald , F. Warburton , C. Robertson , N. B. Pitts , and J. E. Gallagher , “Refocusing Dental Care: A Risk‐Based Preventative Oral Health Programme for Dentate Older People in UK Care Homes,” Gerodontology 39, no. 2 (2022): 131–138. 33586205 10.1111/ger.12543

[12] K. Paddock , C. Brown Wilson , C. Walshe , and C. Todd , “Care Home Life and Identity: A Qualitative Case Study,” Gerontologist 59, no. 4 (2019): 655–664. 30085052 10.1093/geront/gny090 PMC6630159

[13] L. Warren , J. E. Kettle , B. J. Gibson , A. Walls , and P. G. Robinson , “I've Got Lots of Gaps, but I Want to Hang on to the Ones That I Have’: The Age‐Ing Body, Oral Health and Stories of the Mouth,” Ageing and Society 40, no. 6 (2020): 1244–1266.

[14] S. Allan and F. Vadean , “The Association Between Staff Retention and English Care Home Quality,” Journal of Aging & Social Policy 33, no. 6 (2021): 708–724. 33470916 10.1080/08959420.2020.1851349

[15] M. Calvo and M. Sclater , “Creating Spaces for Collaboration in Community Co‐Design,” International Journal of art & Design Education 40, no. 1 (2021): 232–250.

[16] J. Gabbay and A. le May , “Mindlines: Making Sense of Evidence in Practice,” British Journal of General Practice 66, no. 649 (2016): 402–403. 10.3399/bjgp16X686221 PMC4979949 27481961

