# Supplementary material for: A Theoretically Informed Process Evaluation in Parallel to a Feasibility Study of a Complex Oral Health Intervention Using NICE Guidelines in a Care Home Setting
Source: Community Dent Oral Epidemiol. 2025 Jan 15;53(2):152–9. doi: 10.1111/cdoe.13016 (PMC11892547; doi:10.1111/cdoe.13016)
Supplement: Supplementary file 1 — Supporting File S1. [file CDOE-53-152-s001.docx]

**Supporting File**

Additional quotes for each theme are detailed below.

***Paradigm and goals***

All the care home managers interviewed expressed a strong desire to participate in the study, particularly as access to local dental services was difficult.

*You know, I wanted to be part of how we can promote it or what we need to do and what we are not doing I:5.23 (care home manager*)

Equally, participation in the study appeared to have a marked impact on the reported daily routines for care home staff.

*It's not a big ask, it just needs to be [done]. This is what you do. I incorporated into everybody's job [plan], yeah I:9.211 (care home manager)*

*One thing we have found is we're renewing their toothbrushes where we were never doing that before I:8.101 (care home manager)*

***System structure***

The manner of adoption of the study appeared to be relatively idiosyncratic. The researchers reported in their reflexive workshop that the organisation and the efficiency of processes within each home were very different.

*I think I would say every care home is unique. No matter what their organization is, what their ownership is like W:3,019 (researcher 2)*

*We have care homes in the study belonging to the same group [of care homes]. One care home absolutely organized and the care home from the same group…. ….not W:2,864 (researcher 1)*

Equally, certain features of the care home were seen to be more important than others. One key element here was the position and personality of the person in charge of dealing with the TOPIC study at the home.

*When the study started in one of the care homes, the person in charge was just a care home staff, and at the beginning of the study we had, like lots and lots of missing data from there…. ….but when she was promoted everything was very well organized W:3,059 (researcher 1)*

Time poverty was raised as a significant issue in the workshop and in the interviews.

*It takes time to give them [the residents] their personal care and to get them ready W:6.762 (care home staff)*

*There's a lot of staffing issues, if you don't have enough staff, it becomes difficult for them to do it [the intervention] I:4.341 (care home staff)*

Competing needs was another factor raised in the interviews, with care home staff prioritising one task over another due to time poverty and the fluctuating care needs of the residents. The new oral health routine was an additional task in an already busy schedule and this was exacerbated further in homes with a higher proportion of residents requiring nursing care.

*So, they are more happy to give someone a shower and give them breakfast and forget about those other bits, yeah I:1.862 (care home manager)*

Differences between shift patterns, shaped by the routines within the care home was also raised in the interviews and the workshop.

*The night time staff would be getting the residents ready for bed and so they would have [the oral health routine] at the start and at the end of their shifts [morning routine]…. ….it's one of many other jobs they just had to do" I:9.103 (care home manager)*

*They need to brush their teeth before they go to bed. So…. ….doesn't really fit our workload or anything I:2.778 (care home staff)*

Staff turnover was an important factor raised in both the interviews and the workshop.

*There's a staffing issue, if you don't have enough staff, [it] becomes difficult for them to do it, but they try I:4.337 (care home staff)*

Equally, there appeared to be a difference in how permanent and agency staff responded to the study in a number of homes.

*Some of the care homes have more registered nurses. Some are totally reliant on the agency workers W:2608 (researcher 1)*

**Feedback**

This level of the system lens refers to the processes that are used to support stability and the status quo. One key element that arose at this level was the issue of excessive paperwork and some of those interviewed felt that this should have been integrated more with the residents’ Care Plans.

*When they're actually helping with toothbrushing or helping with maintaining the hygiene, I think they felt like they were doing their job. But when they're doing paperwork they feel like they're doing something extra, it is like an add on W:1,687 (researcher 2)*

Equally, many of those interviewed expressed the importance of integrating anything new, like an oral health routine, into the existing rhythm and processes of the care home.

*The kind of rising routine and the sort of the night-time routine W:2,190 (researcher 1)*

*You don't need to get the person up and brush their teeth straight away, you could allow them to have their breakfast or even brush their teeth, have their breakfast and brush their teeth I:9.226 (care home manager)*

Training was also considered to be a key element in maintaining a minimal level of knowledge within the care home in order to deliver an oral health routine. However, care home managers often cited the challenge of training in a care home environment and in the workshop, there were some reports of a ‘tick-box’ attitude towards the training. This aspect was also exacerbated by the level of staff turnover.

*And this stuff [training] was very helpful and useful I:3.185 (care home manager)*

*And most of them are doing it [training] just to tick the boxes for some reason W:3,370 (researcher 2)*

**Structural elements**

Most residents appeared to receive the intervention well, although it was considered important for the care staff to have a good relationship with the resident to facilitate this.

*They only had to be monitored to actually ensure that they were brushing their teeth and brushing them I:9.74 (care home manager)*

*It has been beneficial to the staff and the residents. And because the residents were made to feel as if they're being chosen, it has, you know, highlighted the importance of good oral care I:8.187 (care home manager)*

The cognitive capacity of the resident was also viewed as important.

*And it has to be made enjoyable for it, even if it's something silly like singing a wee song or or not. Not to make them out to sound childish. But some of our residents do have dementia and they enjoy things like that. So it's about making it a fun activity I:8.26 (care home manager)*

*One person that didn’t like his teeth being brushed and he's nonverbal, but he does not like his teeth being brushed I:9.136 (care home manager)*

In the reflexive workshop, cognitive capacity was also reported to fluctuate over the course of the study, which could impact on communication and make it very difficult at times to collect the research data.

*They were mild to moderate [in terms of cognitive impairment] when we recruited them, but their ability declined over the course of the study W:896 (researcher 2)*

Staff attitudes also varied. Some participants suggested that not all staff want to participate in an intervention to improve oral health. There was also some concern relating to the confidence of the care home staff, in terms of determining the difference between oral health and disease.

*What should be the colour of the tongue? So, we know we are not nurses 1.83 (care home manager)*

Some staff felt that the Oral Health Assessment Tool was helpful as it raised the awareness of oral health within the care home.

*I'm getting a bigger picture…. ….I'm getting to understand who actually brushes their own teeth. Who's having a challenge with it? Who needs much more intervention? 1.138 (care home manger*)

However, completing the tool added further burden on the staff and often didn’t change the carer’s behaviour, which was driven by the pressing needs of the resident.

*We got so much paperwork in our folder that we have to do every hour I:2.1,184 (care home staff)*

*The needs of the residents have become so high…. ….they prioritise what they might feel is more urgent I:1.1,264 (care home manger)*

In the workshop, not all the different elements of the TOPIC intervention were reported to be used by all the homes.

*The poster might be helpful because it's on their wall…. ….they would be seeing the poster if they are in the room W:3629 (researcher 2)*

*Only some of them are using those ‘tips and tricks cards’. They don't have time to? They are like stuck at the back of the file W:3,592 (researcher 1)*

*They don't have time to go through the tips and tricks W:3,608 (researcher 2)*
